# Effects of Algal Extracellular Polysaccharides on the Formation of Filamentous Manganese Oxide Particles in the Near-Bottom Layer of Lake Biwa

**DOI:** 10.3390/microorganisms11071814

**Published:** 2023-07-15

**Authors:** Seiko Furuta, Hisato Ikegaya, Megumu Fujibayashi, Hideki Hashimoto, Shiro Suzuki, Kunihiro Okano, Satoshi Ichise, Naoyuki Miyata

**Affiliations:** 1 Lake Biwa Environmental Research Institute, Ohtsu 520-0022, Japan; 2 Department of Biological Environment, Akita Prefectural University, Akita 010-0195, Japan; k_okano@akita-pu.ac.jp; 3 Department of Biology, Kobe University, Kobe 657-0013, Japan; ikegaya16@people.kobe-u.ac.jp; 4 Faculty of Engineering, Kyushu University, Fukuoka 819-0395, Japan; m.fujibayashi@civil.kyushu-u.ac.jp; 5 Department of Applied Chemistry, Kogakuin University, Tokyo 192-0015, Japan; hideki-h@cc.kogakuin.ac.jp; 6 Faculty of Applied Biological Sciences, Gifu University, Gifu 501-1193, Japan; suzuki.shiro.n5@f.gifu-u.ac.jp

**Keywords:** biogenic manganese oxides, manganese(II)-oxidizing bacterium, *Metallogenium*-like particles, total polysaccharide content, transparent exopolymer particles

## Abstract

Filamentous manganese (Mn) oxide particles, which occur in the suboxic zone of stratified waterbodies, are important drivers of diverse elemental cycles. These particles are considered to be bacteriogenic; despite the importance of biogeochemical implications, however, the environmental factor responsible for their formation has not been identified. The aim of this study was to demonstrate the involvement of algal extracellular polysaccharides in Mn oxide particle formation. Based on this study of laboratory cultures of a model Mn(II)-oxidizing bacterium, the supply of algal extracellular mucilage was shown to stimulate Mn(II) oxidation and thus the production of filamentous Mn oxide particles. This observation was consistent with the results obtained for naturally occurring particles collected from a near-bottom layer (depth of approximately 90 m) in the northern basin of Lake Biwa, Japan, that is, most Mn particles resembling δ-MnO_2_ were associated with an extracellular mucilage-like gelatinous matrix, which contained dead algal cells and was lectin-stainable. In the lake water column, polysaccharides produced by algal photosynthesis sank to the bottom layer. The analysis of the quality of water samples, which have been collected from the study site for 18 years, reveals that the annual average total phytoplankton biovolume in the surface layer correlates with the density of filamentous Mn particles in the near-bottom layer. Among different phytoplankton species, green algae appeared to be the key species. The results of this study suggest that algal extracellular polysaccharides serve as an important inducer for the formation of filamentous Mn oxide particles in the near-bottom layer of the northern basin of Lake Biwa.

## 1. Introduction

The manganese (Mn) redox process occurring at the oxic–anoxic interface of stratified waterbodies drives the biogeochemical cycles of numerous elements dissolved in water. Once dissolved Mn(II) ions diffuse from the anoxic layer or sediment to the upper suboxic layer, they are oxidized to form particulate Mn(III, IV) oxides. The Mn oxides serve as scavengers of numerous metal ions and as oxidants of organic and inorganic substances [1,2]. When Mn oxide particles sink to the anoxic layer, they are reduced to Mn(II) ions by an inorganic reductant (i.e., sulfide) or microorganisms with a dissimilatory Mn-reducing activity. Thus, the Mn cycling at the oxic–anoxic interface affects both chemical and biological processes in the water environment (e.g., [3,4,5,6,7,8,9]).

The Mn oxide particles in the oxic layer often have filamentous structures, which are referred to as “*Metallogenium*”-like particles [10,11,12,13,14]. Despite the importance of biogeochemical implications, the particle formation mechanism remains an enigma [15]. The results of a recent study demonstrated that a Mn(II)-oxidizing alphaproteobacterium, *Bosea* sp. BIWAKO-01, produces filamentous Mn particles under laboratory culture conditions [13]. The Mn(II) oxidation and filament formation occurred in static cultures and proceeded faster under CO_2_-rich, low-O_2_ (5–10% in air) conditions. The CO_2_ dependency resulted from the decrease in the cultural pH below 6.0 via carbonation, yielding an optimum pH for Mn(II) oxidation by *Bosea* sp. BIWAKO-01 [13]. Interestingly, a relatively low content of agar (e.g., 500 mg L^−1^) is needed for microbial Mn(II) oxidation. Similar results were reported for other Mn(II)-oxidizing bacteria [16]. It remains unknown whether these bacteria serve as producers of filamentous Mn particles in the water environment. However, the results obtained for these bacterial cultures indicate that environmental factors trigger the bacterial production of particles.

Given that a low concentration of polysaccharides stimulates the Mn(II) oxidation in certain bacterial cultures [13,16], such substances may be responsible for the occurrence of Mn particles in the environment. Diverse phytoplankton species produce extracellular mucilage of acidic polysaccharides [17]. Such gelatinous matter is ubiquitous in the form of transparent exopolymer particles (TEP) in aquatic environments [18]. The results of previous research indicated the significant contribution of TEP to the organic carbon pool in aquatic environments [18,19]. These exopolymers provide microhabitats for colonization by bacteria, leading to high metabolic activity at microparticle surfaces [20,21,22,23]. The effects of phytoplankton or the extracellular polysaccharides on the formation of filamentous Mn particles in stratified waterbodies have not been considered, although laboratory culture experiments [13,16] suggest their contribution.

The results of several studies that were carried out in both the southern and northern basins of Lake Biwa, Japan indicated the presence of filamentous Mn-rich particles in hypoxic layers [24,25,26]. Our working hypothesis is that algal extracellular polysaccharides produced in the epilimnion reach the lower layer by sinking and induce the microbial formation of filamentous Mn particles. In this study, the significance of algal mucilage for Mn particle formation was investigated using laboratory cultures of the Mn(II)-oxidizing bacterium *Bosea* sp. BIWAKO-01. Microscopic analysis was used to determine the structural features of naturally occurring Mn filaments, which were collected at the Imazuoki-chuo point in the northern basin of Lake Biwa. The spatial and temporal distribution of polysaccharides in the water column was monitored at the study site to show that algal polysaccharides were present in the near-bottom layer in which filamentous Mn particles occur. Furthermore, we used water quality data that have been obtained over the past 18 years at the study site to examine whether the particle formation correlates with the growth of phytoplankton in the surface layer. The results of this study support the significance of algal extracellular polysaccharide production.

## 2. Materials and Methods

### 2.1. Culture Experiments

Cultures of a Mn(II)-oxidizing alphaproteobacterium, *Bosea* sp. strain BIWAKO-01, were used as a laboratory model system to yield filamentous Mn particles. Strain BIWAKO-01 was statically cultured in Petri dishes in the dark at 10% O_2_ and 20 °C, as previously described [13]. The M3 liquid culture medium [16] contained 100 mg L^−1^ malt extract, 40 mg L^−1^ yeast extract, 0.5 mM NaHCO_3_, and 500 mg L^−1^ agar. Filter-sterilized MnSO_4_ solution was added to the medium at 0.1 or 2 mM.

To examine the effect of the addition of algal biomass on bacterial Mn(II) oxidation, the 20 mL M3 medium from which agar was omitted was inoculated with BIWAKO-01 at 1.6 × 10^6^ CFU (CFU: colony forming unit), along with green algal species, *Staurastrum arctiscon* or *S. dorsidentiferum*, which were isolated from the water of Lake Biwa and stored at the Lake Biwa Environmental Research Institute (LBERI; Shiga, Japan). These green algae were grown in CT medium [27] (Table S1) at 20 °C using 12 h light–dark cycles. The light intensity was set to 60 µmol m^−2^ s^−1^. After three weeks, the cell densities of *S. arctiscon* and *S. dorsidentiferum* reached 1.6 × 10^4^ and 1.5 × 10^4^ cells mL^−1^, respectively, and 1 mL of these cultures was transferred into 20 mL of BIWAKO-01 cultures. In this experiment, 2 mM MnSO_4_ was added to the culture in the beginning. For the removal of extracellular mucilage sheaths from algal cells, *S. arctiscon* and *S. dorsidentiferum* cells were harvested by filtration using glass fiber filters (Whatman GF/B; GE Healthcare, Buckinghamshire, UK) and then repeatedly washed by injecting sterile distilled water using a spray bottle [28] until the extracellular sheath became invisible under the light microscope when stained with India ink (see below). The cells on each filter were rinsed and resuspended in the CT medium to obtain a cell density of 1.5 × 10^4^ cells mL^−1^. The culture supernatant fluid was removed by centrifugation at 10,000× *g* for 10 min at 4 °C, and the oxidized Mn was determined spectrophotometrically using leucoberbelin blue [29]. For this assay, KMnO_4_ solution was used as standard.

### 2.2. Electron and Light Microscopy

Filamentous Mn oxide particles were filtered through a carbon-coated membrane filter (Nisshin EM, Tokyo, Japan) and vacuum-dried. The particles were analyzed with a transmission electron microscope (TEM; JEM-2100F, JEOL, Tokyo, Japan) at 200 kV and selected area electron diffraction (SAED).

Suspended solids containing gelatinous organic substances were stained with India ink and observed using a differential interference microscope (Eclipse 80i, Nikon, Tokyo, Japan). Under the microscope, transparent gelatinous substances became visible; after staining, they appeared white in contrast to the background. The densities of the filamentous Mn particles in the water samples were measured with the microscope at 100–200× magnification. Lectin staining and fluorescence microscopy were used to detect polysaccharidic substances, as described previously [13,30]. This assay was conducted with a BX60 epifluorescence microscope equipped with a DP70 charge-coupled device camera (Olympus, Tokyo, Japan). The suspended matter including filamentous Mn particles collected in the near-bottom layer of Lake Biwa was gently rinsed with deionized water and stained for 15 min at 23 °C with fluorescein-conjugated Lycopersicon esculentum lectin (LEL), Lens culinaris agglutinin (LCA), Phaseolus vulgaris erythroagglutinin (PHA-E), soy bean agglutinin (SBA), or peanut agglutinin (PNA). These lectins were purchased from Vector Laboratories (Burlingame, CA, USA). Epifluorescence microscopy was conducted at an excitation wavelength of 470 to 490 nm.

### 2.3. Study Site and Available Water Quality Data

Lake Biwa is a warm monomictic lake in the Shiga Prefecture, Japan, with an area of 670 km^2^ and maximum depth of 104 m. The study site was the Imazuoki-chuo point in the northern basin of Lake Biwa (35°23′41″ N, 136°07′57″ E). For the fixed-point study, the water quality was monitored bimonthly by the LBERI at depths of 0.5, 5, 10, 15, 20, 30, 40, 60, 80, 85, and 90 m. Because the water depth in this area is approximately 90 m, the 90 m sample was collected 1 m above the bottom of the lake. We monitored the vertical distribution of polysaccharides at the study site from August 2014 to February 2020. The water samples collected at the above-mentioned depths, except for the 30 m sample, were provided by the LBERI.

To investigate the environmental factors affecting the occurrence of filamentous Mn particles, LBERI’s database was used. The database contains water quality data obtained on samples that were collected twice a month at the study site from April 2002 to February 2020 including the pH and dissolved oxygen (DO), total organic carbon (TOC), total nitrogen (TN), total phosphorus (TP), and chlorophyll *a* (Chl.*a*) contents. These data are partially available on a website [31]. In addition, 18-year data of the abundance of filamentous Mn particles observed at a depth of 90 m and the biovolume of phytoplankton at 0.5 m were used. The total biovolume was expressed as the sum of the biovolumes of Cyanophyceae (cyanobacteria), Chlorophyceae (including Charophyceae; green algae), Bacillariophyceae (diatoms), Chrysophyceae, Dinophyceae, and Cryptophyceae, which were calculated from the cellular or colony sizes, as described elsewhere (Kishimoto et al. 2013). The biovolume data obtained from 2002 to 2009 at the Imazuoki-chuo point were published previously [32,33].

**Figure 1:**
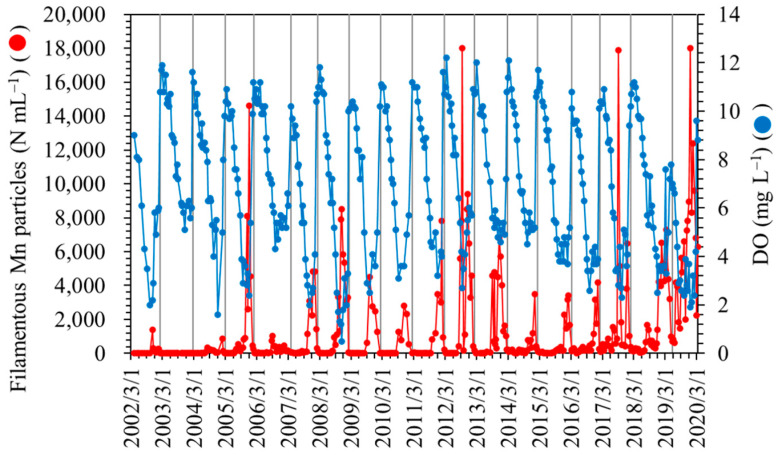
Long-term variations in filamentous Mn particle densities and DO concentrations at a depth of 90 m at the study site. Vertical lines represent complete overturns of the water column.

The water stratification that develops during the summer season at the study site results in the depletion of DO in the near-bottom layer, which is placed under hypoxic or subhypoxic conditions in the fall and winter months (Figure 1). During this period, Mn^2+^ ions are eluted from lake sediments rich in Mn oxides and diffuse to and are reoxidized in the oxic or suboxic near-bottom layer [34]. Subsequently, the stratified water column overturns usually until late winter, as represented by the bottom layer DO content in Figure 1. Therefore, the Mn particle density decreases to a nondetectable level via dispersion. The annual cycle of filamentous Mn particle formation in the near-bottom layer proceeds during the period of the complete overturn to the next complete overturn. In this study, the annual average values of the density of filamentous Mn particles and other water quality parameters were calculated using data obtained during one annual cycle of Mn particle formation (counted from February or March of the present year to the next; Figure 1).

### 2.4. Analysis of Polysaccharides

For the analysis of the total polysaccharides accompanied by free and cell-bound fractions, 250 to 300 mL of the water samples were freeze-dried. The total solids were suspended in 10 mL of methanol to concentrate the contents 25 to 30 times. A portion of the methanol suspension was transferred into a glass tube and dried under a gentle N_2_ stream. The residual solids were then resuspended in 2.0 mL of deionized water. The total polysaccharide concentrations were determined with an anthrone reagent [35]. Briefly, the concentrated suspension (2 mL) in a glass tube was placed in an ice bath for 5 min, and 4 mL of an anthrone reagent containing 0.2 g anthrone in 100 mL of 95% sulfuric acid was slowly added to the glass tube and then mixed vigorously. The glass tube was heated for 10 min using a boiling water bath and allowed to cool at room temperature. The absorbance at 625 nm was determined with a spectrophotometer. The measurements were conducted in triplicate. Glucose solution was used as standard.

The neutral monosaccharide composition was analyzed using a previously described procedure [36]. Briefly, the water samples were freeze-dried, and the solids were hydrolyzed with 4 M trifluoroacetic acid at 100 °C for 3 h. The alditol acetates were separated and quantified using the Development and Assessment of Sustainable Humanosphere system (DASH) at Kyoto University, that is, a gas chromatograph–mass spectrometer system QP2010 (Shimadzu, Kyoto, Japan) equipped with a capillary column (SP-2330, 0.25 mm × 0.2 μm × 15 m; Sigma-Aldrich, St. Louis, MO, USA). The alditol acetates were identified based on their retention times and mass spectra.

### 2.5. Statistical Analysis

Principal component analysis (PCA) was performed to identify the variables that positively correlated with the Mn particles. The annual average values of water quality parameters, including the pH, DO, TOC, TN, TP, and algal volume, were used for the first PCA, and the biovolume of each algal species was utilized for the second PCA. The analysis was conducted using PRIMER 6 software (Primer-E, Plymouth, UK).

## 3. Results

### 3.1. Filamentous Mn Particle Formation in Laboratory Model System

**Figure 2:**
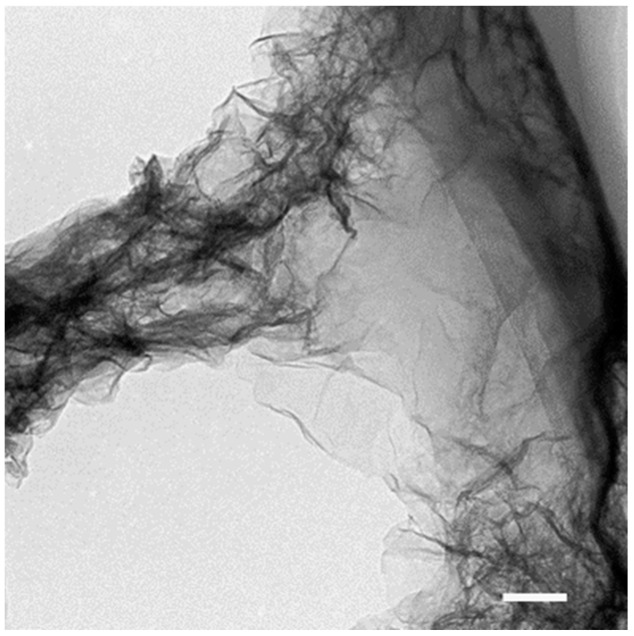
TEM image of filamentous Mn oxide particle collected from BIWAKO-01 culture at day 21. The high-magnification image shows that the filaments have a sheet-type structure. Bar: 100 nm.

The Mn(II)-oxidizing bacterium *Bosea* sp. BIWAKO-01 was cultured in a medium containing agar to which 0.1 mM of dissolved Mn(II) was intermittently added for 14 days to obtain a total dose of 0.7 mM (Figure S1). The cumulative formation of oxidized Mn due to the addition of Mn(II) indicates that the dissolved Mn(II) ions in the medium are mostly oxidized and maintained at low concentrations. The TEM observations reveal that the filamentous particles consist of nano-sized sheets, which are consistent with the structure of the biogenic layer-type Mn oxide mineral δ-MnO_2_ (Figure 2).

**Figure 3:**
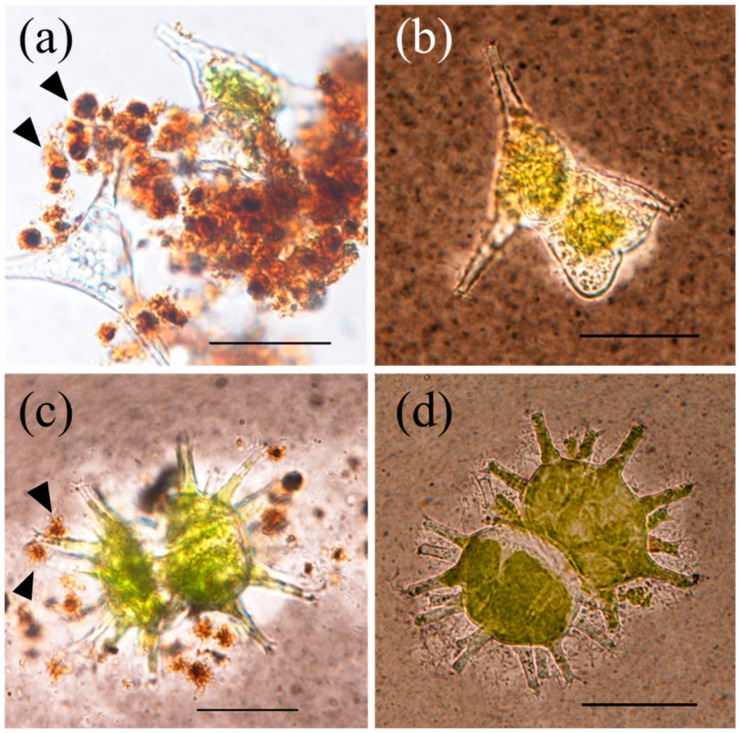
Microscopic images of filamentous Mn oxide particles that formed in laboratory cultures of *Bosea* sp. BIWAKO-01. The bacterial strain was grown in the presence of green algae: *S. dorsidentiferum* intact (**a**) and mucilage-sheath-removed (**b**) cells; *S. arctiscon* intact (**c**) and mucilage-sheath-removed (**d**) cells. Images were obtained from India-ink-stained specimens. The arrowheads indicate filamentous Mn particles. Bar: 30 μm.

The results showed that the strain BIWAKO-01 cannot oxidize Mn(II) in the absence of agar during the culture period, as previously reported [13]. However, this strain oxidized Mn(II) in the agar-free medium when supplemented with a green algal species, that is, *S. dorsidentiferum* or *S. arctiscon*. When Mn(II) was added at 2 mM, the oxidized Mn content reached 1.2 ± 0.3 and 0.7 ± 0.2 mM (mean ± standard deviation; *n* = 3) in the presence of *S. dorsidentiferum* and *S. arctiscon*, respectively (Figure 3). A large proportion of the Mn particles was in localized contact with extracellular mucilage sheaths of algal cells, which appeared white in contrast to the background after India ink staining. The content of oxidized Mn was below the detection level (<0.01 mM) in the cultures with naked algal cells whose extracellular mucilage sheaths were extensively removed by spraying with deionized water. Filamentous particles were not observed in these cultures, demonstrating the significance of extracellular mucilage produced by algal cells for the Mn(II) oxidation and production of filamentous Mn particles by the bacterium.

### 3.2. Filamentous Mn Particles Collected in the Near-Bottom Layer of Lake Biwa

**Figure 4:**
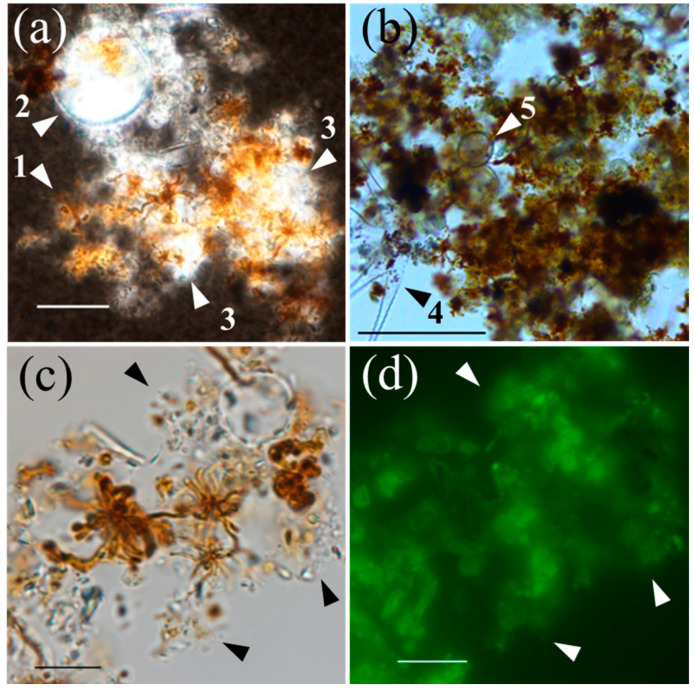
Microscopic images of filamentous Mn oxide particles collected at a depth of 90 m at the study site. (**a**) India-ink-stained aggregates including filamentous Mn particles (arrowhead 1), dead algal cells (arrowhead 2), and gelatinous substances (arrowhead 3). Bar: 20 μm. (**b**) Unstained aggregates including numerous Mn particles and dead algal cells (arrowheads 4 and 5). Bar: 100 μm. (**c**,**d**) Differential interference contrast and epifluorescence images of aggregates stained with fluorescein-conjugated LEL. Gelatinous substances are indicated with arrowheads. Bar: 10 μm.

The sizes of naturally occurring filamentous particles collected from the near-bottom layer ranged from approximately 5 to 20 μm (Figure 4). The particle suspensions reacted with leucoberbelin blue, indicating the presence of oxidized Mn. The TEM and SAED analyses reveal that the Mn mineral is δ-MnO_2_ with *d* values of 0.253 and 0.148 nm (Figure S2). The filamentous particles coexisted with aggregates including fine solids and gelatinous substances, which became visible after India ink staining (Figure 4). Several dead cells of green algae and diatoms (frustules) were observed in the aggregates. Staining with various lectins, including LEL (recognizing *N*-acetylglucosamine; Figure 4), LCA (α-mannose and α-glucose; Figure S3), PHA-E and SBA (*N*-acetylgalactosamine), and PNA (galactose), indicated that the organic matrix of the aggregates contains these polysaccharidic moieties. These lectins also bind to the filaments.

### 3.3. Distribution of Polysaccharides in the Water Column of Lake Biwa

**Figure 5:**
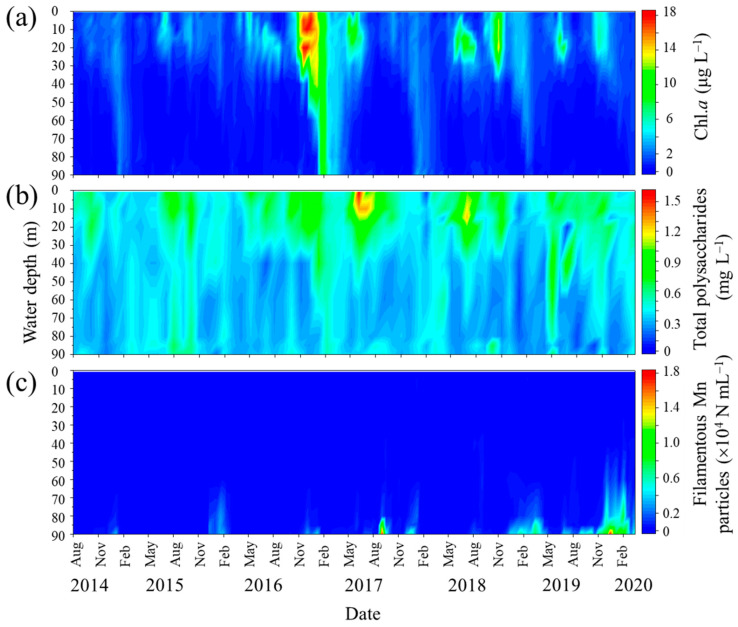
Seasonal and vertical variations in Chl.*a* (**a**) and total polysaccharide (**b**) concentrations and filamentous Mn particle densities (**c**) at the study site.

**Figure 6:**
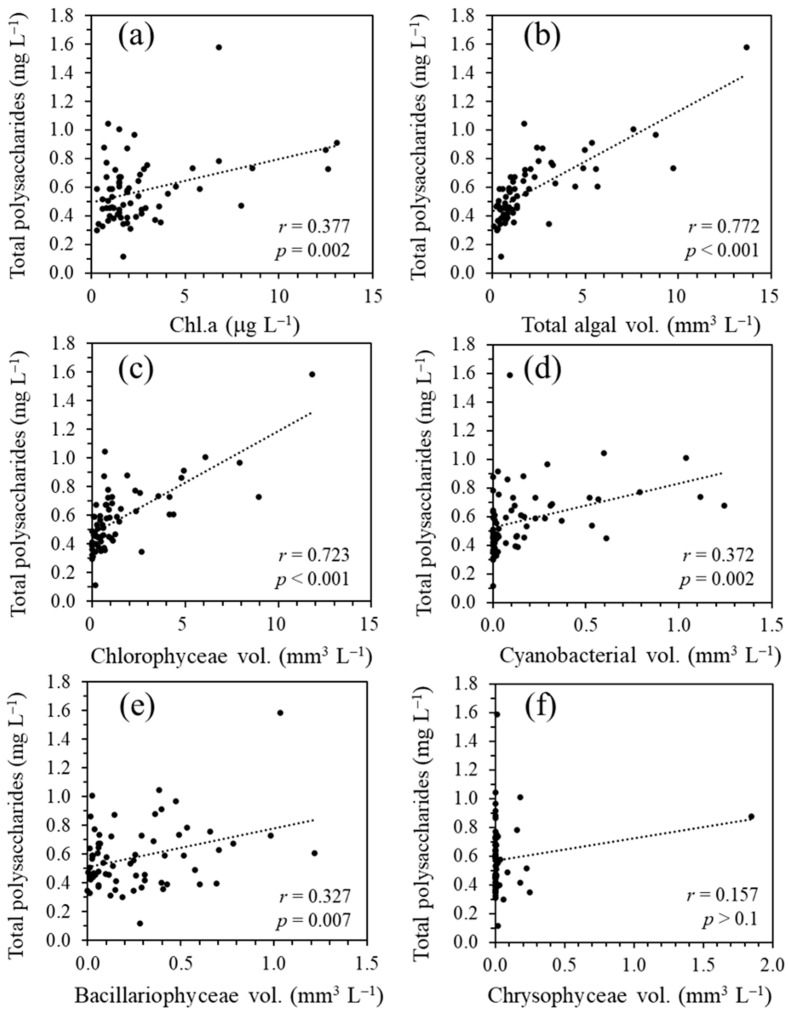
Correlations between the total polysaccharide and Chl.*a* concentrations (**a**), total phytoplankton biovolume (**b**), *Chlorophyceae biovolume* (**c**), *Cyanobacteria biovolume* (**d**), Bacillariophyceae biovolume (**e**), and *Chrysophyceae biovolume* (**f**). All the data were obtained at a depth of 0.5 m at the study site of Lake Biwa.

The spatiotemporal distribution of the total polysaccharides in the water column was monitored from August 2014 to March 2020. The data are shown in Figure 5 along with the Chl.*a* concentrations and filamentous Mn particle densities derived from LBERI’s database. During the study period, the total polysaccharide concentration at a depth of 0.5 m was 0.51 ± 0.15 mg L^−1^ (*n* = 14) in the spring season (March to May), 0.76 ± 0.29 mg L^−1^ (*n* = 16) in the summer season (June to August), 0.60 ± 0.12 mg L^−1^ (*n* = 18) in the fall season (September to November), and 0.45 ± 0.18 mg L^−1^ (*n* = 18) in the winter season (December to February). From August 2014 to February 2020, the total polysaccharide concentration at a depth of 0.5 m strongly correlated with the biovolume of phytoplankton cells (*r* = 0.772, *p* < 0.001; Figure 6) and was less but still significantly correlated with the Chl.*a* concentration (*r* = 0.377, *p* = 0.002). Among the phytoplankton species, green algae were the most dominant with respect to the total biovolume and significantly correlated with the total polysaccharides (*r* = 0.723, *p* < 0.001; Figure 6), suggesting that these species mainly contribute to the production of polysaccharides in the surface layer. The profiles in Figure 5 also show that a certain part of the polysaccharides in the surface layer sinks to the near-bottom layer. During the monitoring term, the total polysaccharide concentration at a depth of 90 m ranged from 0.17 to 0.64 mg L^−1^ (0.36 ± 0.10 mg L^−1^; *n* = 68).

The total polysaccharides obtained at depths of 0.5 and 90 m included rhamnose, fucose, arabinose, xylose, mannose, galactose, and glucose (Figure S4). The compositions of these saccharides temporally changed but insignificantly differed spatially, although two 90 m samples from July 2015 and December 2016 contained higher glucose concentrations. In general, the saccharide compositions at 0.5 m were similar to those at 90 m. Significant correlations were observed for rhamnose (*r* = 0.561; *p* = 0.001), fucose (*r* = 0.456; *p* = 0.010), arabinose (*r* = 0.360; *p* = 0.046), xylose (*r* = 0.797; *p* < 0.001), and mannose (*r* = 0.503; *p* = 0.004).

Typically, the densities of filamentous Mn particles in the near-bottom layer are high from late fall to winter due to the DO depletion (Figure 1 and Figure 5). This period is inconsistent with the period during which high total polysaccharide concentrations were observed in the bottom layer (Figure 5). The correlation between the density of filamentous Mn particles and the total polysaccharide concentration at 90 m was insignificant (*p* > 0.1).

### 3.4. Correlation between Filamentous Mn Particles and the Phytoplankton Biovolume in the Past 18 Years

**Figure 7:**
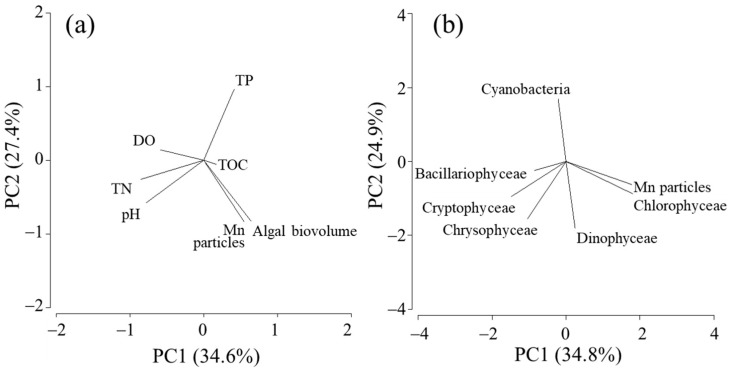
PCA biplot for the annual average values of filamentous Mn particle densities and water quality data (**a**) and biovolume of algal species (**b**) collected over 18 years at the study site.

**Figure 8:**
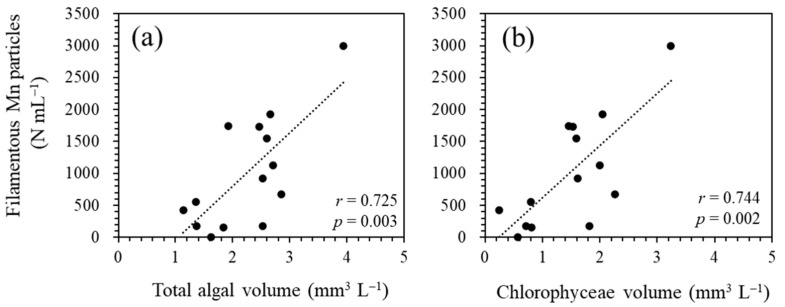
Correlations between the filamentous Mn particle density at 90 m and total phytoplankton biovolume (**a**) and Chlorophyceae biovolume (**b**) at 0.5 m. The plots represent the annual average data collected for 18 years.

Among the 18-year water quality data, three periods (i.e., March 2009–February 2010, March 2010–February 2011, and February 2013–February 2014; Figure 1) were excluded from the calculation because of the lack of more than three data points during the emergence of filamentous Mn particles. The period June 2019–March 2020 was also excluded because of the incomplete overturn of the water column (Figure 1), which resulted in the chronic formation of particles in the near-bottom layer. The PCA of annual average data (*n* = 14; Figure 7) reveals the relationship between the density of filamentous Mn particles at 90 m and the total algal biovolume at 0.5 m. Among the different algal species, this relationship was observed for the biovolume of green algae (Figure 7). The coefficients of the correlations between Mn particles and the total algal biovolume and green algal biovolume were determined to be 0.725 (*p* = 0.003) and 0.744 (*p* = 0.002), respectively (Figure 8). These results are consistent with the contribution of phytoplankton (green algae) in the surface layer to the formation of filamentous Mn particles. The correlations with other water quality parameters, including the pH, DO, TOC, TN, and TP at 90 m, were insignificant.

## 4. Discussion

The results of a previous study of *Bosea* sp. BIWAKO-01 cultures demonstrated that the addition of a low concentration of gelatinous polysaccharides, such as agar and agarose, stimulates the bacterial oxidation of Mn(II) [13]. In the presence of polysaccharides, the BIWAKO-01 cell extends a few filaments from the cell surface and becomes encapsulated with the increase in the number of filaments. Oxidized Mn phases include γ-MnOOH, a needle-type Mn(III) oxide [13], which likely forms via the reduction of thin sheet-like δ-Mn(IV)O_2_ by excess dissolved Mn^2+^ ions. Thus, the dissolved Mn^2+^ remaining in the culture fluid controls the mineralogy of the final product [37]. In this study, a low concentration of dissolved Mn^2+^ (0.1 mM) was added intermittently, which hindered the formation of Mn(III) oxide, maintaining the sheet-like structures of δ-MnO_2_ (Figure S1 and Figure 2). Filamentous Mn particles from Lake Biwa had a similar structure to that of δ-MnO_2_ (Figure S2), as widely reported for biogenic or naturally occurring Mn oxide phases [1,2].

The addition of polysaccharides to BIWAKO-01 cultures might be relevant to the static conditions affecting the cellular motility in a slightly viscous medium or polymeric matrix [13], although its role remains unknown. In this study, a similar effect was observed for extracellular mucilage-bearing cells of green algae, *S. dorsidentiferum* and *S. arctiscon* (Figure 3). Diverse microalgae, including green algae, diatoms, and cyanobacteria, nonenzymatically and enzymatically oxidize Mn(II) [38,39,40,41]. The results of this study show that the Mn(II) oxidation depends on the presence of BIWAKO-01. Green algae could not oxidize Mn(II) in cultures without BIWAKO-01. The Mn oxide particles were in localized contact with the mucilage matrix (Figure 3), suggesting that BIWAKO-01 cells grow on the matrix and produce the Mn oxide particles. This phenomenon is consistent with observations made on natural Mn particles in the near-bottom layer of Lake Biwa, which are in contact with organic aggregates containing gelatinous, lectin-stainable substances, and dead algal cells (Figure 4 and Figure S3). The reactivity of the filaments during staining with the lectins PHE-A, SBA, and PNA was relatively high, suggesting that the filaments also contain lectin-stainable polysaccharides. This was the case for the filaments produced by BIWAKO-01; the filament structures consist of lectin-stainable acidic polysaccharides and Mn oxide phases [13]. Our observations are similar to those of previous studies. In the hypolimnion of a freshwater reservoir, filamentous Mn particles were present in a mixture of algae, diatoms, and amorphous material [42]. The results of several studies demonstrated the presence of Mn precipitates associated with diatom frustules in freshwater biofilms [43,44]. In the Gotland Basin, Baltic Sea, 68% of all Mn-rich particles observed in the stratified column were associated with large organic aggregates with radii ranging from 10 to 54 μm [5]. The Mn-particle-associated polysaccharidic substances are reported to serve as microbial hotspots and affect their stationary sinking velocity by altering the particle density [45]. The results of the present study suggest the significant contribution of such organic material to the biogenic formation of Mn oxide particles.

The presence of dead algal cells in the near-bottom layer (Figure 4) indicates that photosynthetic products, including algal biomass, sink to the lake bottom. At the study site, the distribution of total polysaccharides in the water column was widespread but varied spatiotemporally (Figure 5). The higher concentration gradient in the upper layer and correlations with the biovolume of phytoplankton cells or Chl.*a* concentration indicate that these substances originate from phytoplankton in the upper layer (Figure 6). Green algae are considered to be responsible for the total polysaccharide concentration at the study site. In an oligotrophic reservoir, the TEP produced in the surface layer contribute to the carbon export to the hypolimnion at 30 m, with values of 0.02% to 31% [46]. Although our observations provide indirect evidence for the origin of gelatinous polysaccharides in the near-bottom layer, the decomposition, utilization, and transformation of photosynthetic products by heterotrophic microorganisms during sinking cannot be excluded, as previously reported [18,22,47]. The results of a previous study in the northern basin of Lake Biwa showed that HCl-hydrolyzable saccharides in the epilimnion (2.5 m) and hypolimnion (40 m) consist of relatively large quantities of rhamnose, fucose, arabinose, and xylose [48]. This is consistent with our observations (Figure S4). The high activity of heterotrophic consumers in the lake water observed in previous studies suggests that polysaccharides, which are photosynthetically produced in the epilimnion, are modified by the heterotrophs, and the remaining refractory polysaccharides reach the hypolimnion [48,49].

Although the results obtained for laboratory microbial cultures suggest the involvement of algal extracellular polysaccharides in the formation of filamentous Mn particles (Figure 3), the variation in filamentous Mn particles in the near-bottom layer cannot be explained by the total polysaccharide concentration. This might result from the different timing of the DO depletion and elevation of polysaccharides in the environment. Therefore, we assume that lectin-positive gelatinous substances are supplied by the sinking of phytoplankton cells throughout the year and remain, at least partly, on the bottom. We examined the correlations among water quality data based on average values obtained during an annual cycle of filamentous Mn particle formation. The analysis of 18-year water quality data reveals a positive correlation between the annual average density of filamentous Mn particles at 90 m and total algal (or green algal) biovolume at 0.5 m (Figure 7 and Figure 8). It is likely that a higher primary production leads to an increase in the DO consumption by heterotrophic consumers in the hypolimnion, accelerating the Mn particle formation. However, the abundance of Mn particles cannot be explained by the fluctuation of the DO or TOC concentration. This suggests that the (green) algal biomass directly contributes to the formation of Mn particles in the near-bottom layer.

Regarding the long-term trend of the total biovolume of phytoplankton cells in the northern basin of Lake Biwa (0.5 m), research showed that the average total biovolume ranged from 1.5 to 1.8 mm^3^ L^−1^ from 2000 to 2009, corresponding to 63% to 85% of the levels observed from 1980 to 1989 [33]. Although the total biovolume decreases, the total volume of mucilage sheaths surrounding the phytoplankton cells (16 to 21 mm^3^ L^−1^ during 2000 and 2009) reaches more than twice the levels of 1980–1989 and 1990–1999, suggesting an increase in phytoplankton species with increasing sheath content. Such a long-term succession of phytoplankton species likely affects the supply of polysaccharides to the near-bottom layer and thus the Mn cycle. In recent years, Lake Biwa has experienced incomplete overturns of the water column by lake warming [31]. It has been argued that the resultant DO depletion seriously affects the fate of Mn and other elements such as arsenic in the bottom environment [34,50]. It has been shown that the redox cycle of Mn in Lake Biwa affects the dynamics of metal ions [34,51,52]. Thus, the phytoplankton succession may interfere with the DO-depletion-induced dynamics of Mn and other metal ions in the bottom environment because algal extracellular polysaccharides might force back metal ions dissolved in water via the induction of microbial Mn oxide formation.

## 5. Conclusions

In this study, we provide several lines of evidence that algal extracellular polysaccharide production is a key environmental factor directly inducing the formation of filamentous Mn oxide particles in the near-bottom layer of the northern basin of Lake Biwa. The laboratory culture experiment and field data analysis suggest that the extracellular polysaccharides induce bacterial Mn(II) oxidation in the environment. Such a polysaccharide-dependent mechanism may be ubiquitous in nature because Mn particles associated with an algal mucilage-like gelatinous matrix have been reported elsewhere. Further studies are needed to clarify the molecular mechanism of filamentous Mn oxide particle formation by Mn(II)-oxidizing bacteria in the presence of polysaccharidic substances.

## Data Availability

All datasets generated for this study are included in the article.

## Supplementary Materials

The following supporting information can be downloaded at: https://www.mdpi.com/article/10.3390/microorganisms11071814/s1, Figure S1: Mn(II) oxidation by *Bosea* sp. BIWAKO-01 in agar-containing cultures; Figure S2: TEM image of filamentous Mn oxide particle collected at a depth of 90 m at the study site of Lake Biwa; Figure S3: differential interference contrast and epifluorescence images of aggregates collected at a depth of 90 m at the study site of Lake Biwa; Figure S4: neutral monosaccharides detected in the total polysaccharides collected from the lake waters at 0.5 m and 90 m during 2014–2017; Table S1: composition of CT medium.

## References

[1] Tebo B.M., Bargar J.R., Clement B.G., Dick G.J., Murray K.J., Parker D., Verity R., Webb S.M. (2004). Biogenic manganese oxides: Properties and mechanisms of formation. Annu. Rev. Earth Planet Sci..

[2] Huang Y., Huangfu X., Ma C., Liu Z. (2023). Sequestration and oxidation of heavy metals mediated by Mn(II) oxidizing microorganisms in the aquatic environment. Chemosphere.

[3] Lienemann C.P., Taillefert M., Perret D., Gaillard J.F. (1997). Association of cobalt and manganese in aquatic systems: Chemical and microscopic evidence. Geochim. Cosmochim. Acta.

[4] Taillefert M., Macgregor B.J., Gaillard J.F., Lienemann C.P., Stahl D.A. (2002). Evidence for a dynamic cycle between Mn and Co in the water column of a stratified lake. Environ. Sci. Technol..

[5] Neretin L.N., Pohl C., Jost G., Leipe T., Pollehne F. (2003). Manganese cycling in the Gotland Deep, Baltic Sea. Mar. Chem..

[6] Dellwig O., Leipe T., März C., Glockzin M., Pollehne F., Schnetger B., Yakushev E.V., Böttcher M.E., Brumsack H.J. (2010). A new particulate Mn−Fe−P-shuttle at the redoxcline of anoxic basins. Geochim. Cosmochim. Acta.

[7] Bauer S., Blomqvist S., Ingri J. (2017). Distribution of dissolved and suspended particulate molybdenum, vanadium, and tungsten in the Baltic Sea. Mar. Chem..

[8] Ossa O.F., Hofmann A., Wille M., Spangenberg J.E., Bekker A., Poulton S.W., Eickmann B., Schoenberg R. (2018). Aerobic iron and manganese cycling in a redox-stratified Mesoarchean epicontinental sea. Earth Planet Sci. Lett..

[9] Henkel J.V., Schulz-Vogt H.N., Dellwig O., Pollehne F., Schott T., Meeske C., Beier S., Jürgens K. (2022). Biological manganese-dependent sulfide oxidation impacts elemental gradients in redox-stratified systems: Indications from the Black Sea water column. ISME J..

[10] Gregory E., Perry R.S., Staley J.T. (1980). Characterization, distribution, and significance of *Metallogenium* in Lake Washington. Microb. Ecol..

[11] Maki J.S., Tebo B.M., Palmer F.E., Nealson K.H., Staley J.T. (1987). The abundance and biological activity of manganese-oxidizing bacteria and *Metallogenium*-like morphotypes in Lake Washington, USA. FEMS Microbiol. Ecol..

[12] Sternbeck J. (1996). Manganese cycling in a eutrophic lake−rates and pathways. Aquat. Geochem..

[13] Furuta S., Ikegaya H., Hashimoto H., Ichise S., Kohno T., Miyata N., Takada J. (2015). Formation of filamentous Mn oxide particles by the alphaproteobacterium *Bosea* sp. strain BIWAKO-01. Geomicrobiol. J..

[14] Zakharova Y.R., Parfenova V.V., Granina L.Z., Kravchenko O.S., Zemskaya T.I. (2010). Distribution of iron- and manganese-oxidizing bacteria in the bottom sediments of Lake Baikal. Inland Water Biol..

[15] Ehrlich H.L., Newman D.K. (2009). Geomicrobiology of manganese. Geomicrobiology.

[16] Miyajima T. (1992). Production of *Metallogenium*-like particles by heterotrophic manganese-oxidizing bacteria collected from a lake. Arch. Microbiol..

[17] Leppard G.G. (1995). The characterization of algal and microbial mucilages and their aggregates in aquatic ecosystems. Sci. Total Environ..

[18] Passow U. (2002). Transparent exopolymer particles (TEP) in aquatic environments. Prog. Oceanogr..

[19] Mari X., Passow U., Migon C., Burd A.B., Legendre L. (2017). Transparent exopolymer particles: Effects on carbon cycling in the ocean. Prog. Oceanogr..

[20] Berman T., Viner-Mozzini Y. (2001). Abundance and characteristics of polysaccharide and proteinaceous particles in Lake Kinneret. Aquat. Microb. Ecol..

[21] Azam F., Malfatti F. (2007). Microbial structuring of marine ecosystems. Nat. Rev. Microbiol..

[22] Callieri C., Corno G., Contesini M., Fontaneto D., Bertoni R. (2017). Transparent expolymer particles (TEP) are driven by chlorophyll a and mainly confined to the euphotic zone in a deep subalpine lake. Inland Waters.

[23] Cai Y.-M. (2020). Non-surface attached bacterial aggregates: A ubiquitous third lifestyle. Front. Microbiol..

[24] Miyajima T. (1992). Biological manganese oxidation in a lake. I. Occurrence and distribution of *Metallogenium* sp. and its kinetic properties. Arch. Hydrobiol..

[25] Furuta S., Yoshida M., Okamoto T., Wakabayashi T., Ichise S., Aoki S., Kono T., Miyajima T. (2007). Morphological variations of a manganese-oxidizing microorganism *Metallogenium* observed in the developmental process of cultures collected from Lake Biwa waters. Jpn. J. Limnol..

[26] Ishikawa K., Furuta S., Nakajima T., Kawanabe H., Nishino M., Maehata M. (2012). Microbes as indicator species in low oxygen environments. Lake Biwa. Interactions between Nature and People.

[27] Watanabe M., Ichimura T. (1977). Fresh- and salt-water forms of *Spirulina platensis* in axenic cultures. Bull. Jpn. Soc. Phycol..

[28] Giroldo D., Vieira A.A.H., Paulshn B.S. (2005). Microbial degradation of extracellular polysaccharides released by a tropical strain of *Staurastrum orbiculare* (Zygnematophyceae). Phycologia.

[29] Boogerd F.C., de Vrind J.P.M. (1987). Manganese oxidation by *Leptothrix discophora*. J. Bacteriol..

[30] Ikegaya H., Nakase T., Iwata K., Tsuchida H., Sonobe S., Shimmen T. (2012). Studies on conjugation of *Spirogyra* using monoclonal culture. J. Plant Res..

[31] Shiga Prefecture (2023) Annual Reports on the Environment. Shiga Prefecture, Japan. https://www.pref.shiga.lg.jp/ippan/kankyoshizen/kankyou/11319.html.

[32] Kishimoto N., Ichise S., Suzuki K., Yamamoto C. (2013). Analysis of long-term variation in phytoplankton biovolume in the northern basin of Lake Biwa. Limnology.

[33] Ichise S., Ikegaya H., Furuta S., Fujiwara N., Ikeda S., Kishimoto N., Nishimura O. (2013). Analysis of long-term variation of phytoplankton biovolume and gelatinous sheath volume in Lake Biwa. Jpn. J. Water Treat. Biol..

[34] Nakashima Y., Shimizu A., Maruo M., Sohrin Y. (2016). Trace elements influenced by environmental changes in Lake Biwa: I Seasonal variations under suboxic hypolimnion conditions during 2007 and 2009. Limnology.

[35] Scott T.A., Melvin E.H. (1953). Determination of dextran with anthrone. Anal. Chem..

[36] Hayashi T., Linskens H.F., Jackson J.F. (1989). Measuring β-glucan deposition in plant cell walls. Plant Fibers. Modern Methods of Plant Analysis.

[37] Lefkowitz J.P., Rouff A.A., Elzinga E.J. (2013). Influence of pH on the reductive transformation of birnessite by aqueous Mn(II). Environ. Sci. Technol..

[38] Richardson L.L., Aguilar C., Nealson K.H. (1988). Manganese oxidation in pH and O_2_ microenvironments produced by phytoplankton. Limnol. Oceanogr..

[39] Knauer K., Jabusch T., Sigg L. (1999). Manganese uptake and Mn(II) oxidation by the alga *Scenedesmus subspicatus*. Aquat. Sci..

[40] Wang R., Wang S., Tai Y., Tao R., Dai Y., Guo J., Yang Y., Duan S. (2017). Biogenic manganese oxides generated by green algae *Desmodesmus* sp. WR1 to improve bisphenol A removal. J. Hazard Mater..

[41] Chaput D.L., Fowler A.J., Seo O., Duhn K., Hansel C.M., Santelli C.M. (2019). Mn oxide formation by phototrophs: Spatial and temporal patterns, with evidence of an enzymatic superoxide-mediated pathway. Sci. Rep..

[42] Stein L.Y., Jones G., Alexander B., Elmund K., Wright-Jones C., Nealson K.H. (2002). Intriguing microbial diversity associated with metal-rich particles from a freshwater reservoir. FEMS Microbiol. Ecol..

[43] Robbins E.I., Corley T.L. (2005). Microdynamics and seasonal changes in manganese oxide epiprecipitation in Pinal Creek, Arizona. Hydrobiologia.

[44] Keim C.N., Nalini H.A., de Lena J.C. (2015). Manganese oxide biominerals from freshwater environments in Quadrilatero Ferrifero, Minas Gerais, Brazil. Geomicrobiol. J..

[45] Glockzin M., Pollehne F., Dellwig O. (2014). Stationary sinking velocity of authigenic manganese oxides at pelagic redoxclines. Mar. Chem..

[46] De Vicente I., Ortega-Retuerta E., Romera O., Morales-Baquero R., Reche I. (2009). Contribution of transparent exopolymer particles to carbon sinking flux in an oligotrophic reservoir. Biogeochemistry.

[47] Ortega-Retuerta E., Duarte C.M., Reche I. (2010). Significance of bacterial activity for the distribution and dynamics of transparent exopolymer particles in the Mediterranean Sea. Microb. Ecol..

[48] Hayakawa K. (2004). Seasonal variations and dynamics of dissolved carbohydrates in Lake Biwa. Org. Geochem..

[49] Urabe J., Nakanishi M., Kawabata K. (1995). Contribution of metazoan plankton to the cycling of nitrogen and phosphorus in Lake Biwa. Limnol. Oceanogr..

[50] Sohrin Y., Nakashima Y., Maruo M. (2016). Trace elements influenced by environmental changes in Lake Biwa: II. Chemical variations in the hypolimnion over the last half-century. Limnology.

[51] Takamatsu T., Kawashima M., Koyama M. (1985). The role of Mn^2+^-rich hydrous manganese oxide in the accumulation of arsenic in lake sediments. Water Res..

[52] Sugiyama M., Hori T., Kihara S., Matsui M. (2005). Geochemical behavior of trace elements in Lake Biwa. Limnology.

