# Supplementary material for: Effects of Algal Extracellular Polysaccharides on the Formation of Filamentous Manganese Oxide Particles in the Near-Bottom Layer of Lake Biwa
Source: Microorganisms. 2023 Jul 15;11(7):1814. doi: 10.3390/microorganisms11071814 (PMC10386369; doi:10.3390/microorganisms11071814)
Supplement: Supplementary file 1 [file microorganisms-11-01814-s001.zip › microorganisms-2487882-SI.pdf]

Effects of Algal Extracellular Polysaccharides on the Formation of Filamentous Manganese Oxide Particles in the near-Bottom Layer of Lake Biwa

Seiko Furuta [1,2], Hisato Ikegaya [3], Megumu Fujibayashi [4], Hideki Hashimoto [5], Shiro Suzuki [6], Kunihiro Okano [2], Satoshi Ichise [1] and Naoyuki Miyata [2,*]

1. Lake Biwa Environmental Research Institute, Ohtsu 520-0022, Japan; furuta-3@ex.bw.dream.jp (S.F.); ichise_home0115@kxf.biglobe.ne.jp (S.I.)
2. Department of Biological Environment, Akita Prefectural University, Akita 010-0195, Japan; k_okano@akita-pu.ac.jp
3. Department of Biology, Kobe University, Kobe 657-0013, Japan; ikegaya16@people.kobe-u.ac.jp
4. Faculty of Engineering, Kyushu University, Fukuoka 819-0395, Japan; m.fujibayashi@civil.kyushu-u.ac.jp
5. Department of Applied Chemistry, Kogakuin University, Tokyo 192-0015, Japan; hideki-h@cc.kogakuin.ac.jp
6. Faculty of Applied Biological Sciences, Gifu University, Gifu 501-1193, Japan; suzuki.shiro.n5s@f.gifu-u.ac.jp

* Correspondence: nmiyata@akita-pu.ac.jp; Tel.: +8118 872 1660

Table S1. Composition of CT medium [27]

	Concentration (L^{-1})
$Ca(NO_3)_2 \cdot 4H_2O$	15 mg
KNO_3	10 mg
$MgSO_4 \cdot 7H_2O$	4 mg
Disodium β-glycerophosphate	5 mg
Thiamin hydrochloride	1 µg
Cyanocobalamin	0.01 µg
Biotin	0.01 µg
TAPS	10 mg
PIV metal salts soln.*	0.3 mL

*This solution contained (per liter) 19.6 mg of $FeCl_3 \cdot 6H_2O$, 3.6 mg of $MnCl_2 \cdot 4H_2O$, 2.2 mg of $ZnCl_2 \cdot 7H_2O$, 0.4 mg of $CoCl_2 \cdot 6H_2O$, 0.25 mg of $Na_2MoO_4 \cdot 2H_2O$, and 100 mg of $Na_2EDTA \cdot 2H_2O$ (pH 8.2).

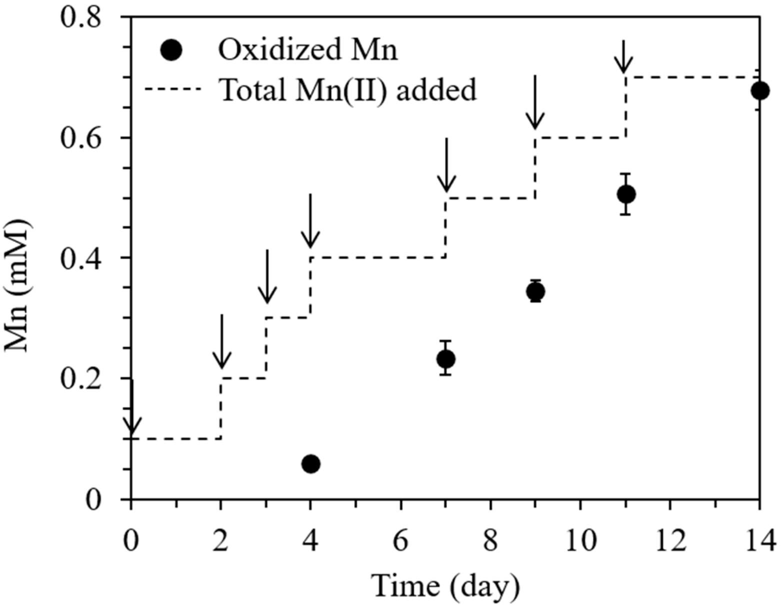

Figure S1. Mn(II) oxidation by *Bosea* sp. BIWAKO-01 in agar-containing cultures. Mn(II) ions were added at a concentration of 0.1 mM at the time indicated by the arrow. Data from triplicate cultures are represented as means ± SD.

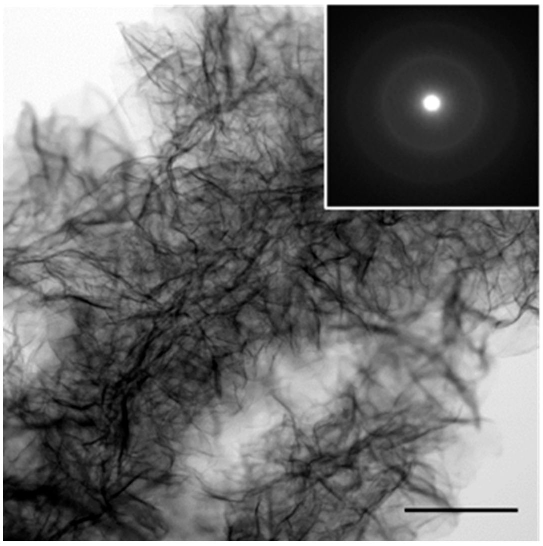

Figure S2. TEM image of filamentous Mn oxide particle collected at a depth of 90 m at the study site of Lake Biwa. The high magnification image shows that the filaments consist of sheet-type structure. Bar: 500 nm. Inset, SAED pattern obtained for the sheet-type structure with *d* values of 0.253 and 0.148 nm.

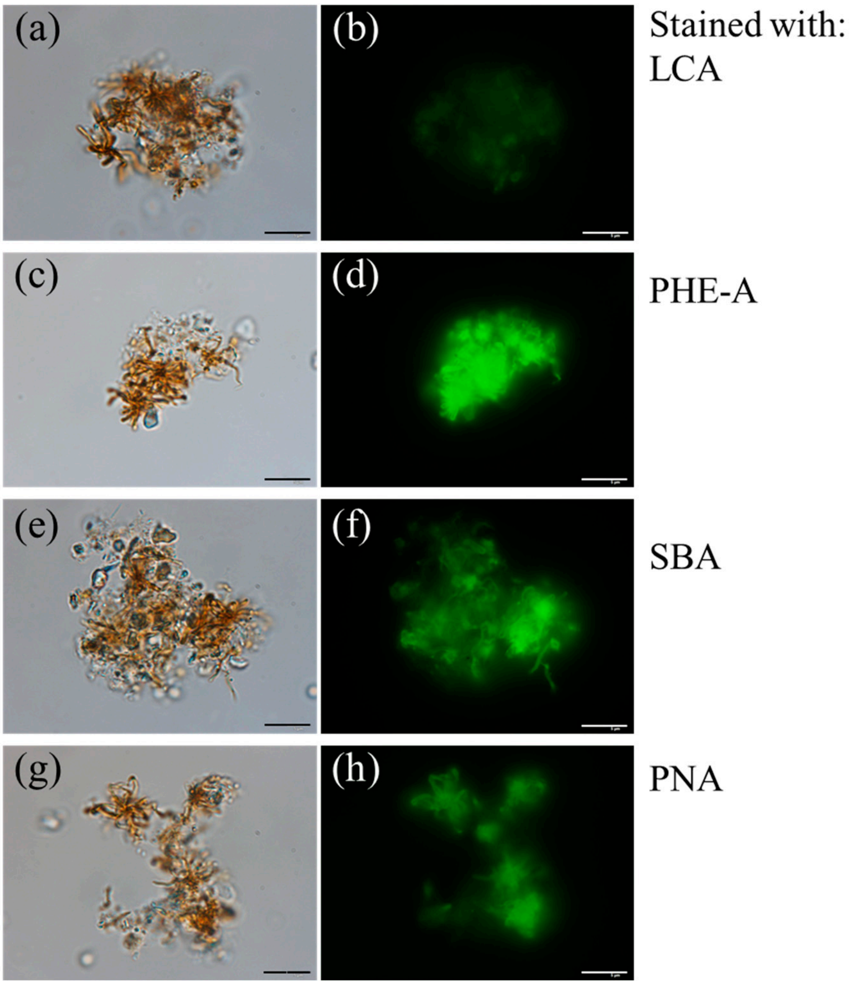

Figure S3. Differential interference contrast and epifluorescence images of aggregates collected at a depth of 90 m at the study site of Lake Biwa. The aggregates were stained with fluorescein-conjugated LCA (a, b), PHA-E (c, d), SBA (e, f), and PNA (g, h). Bar: 10 µm.

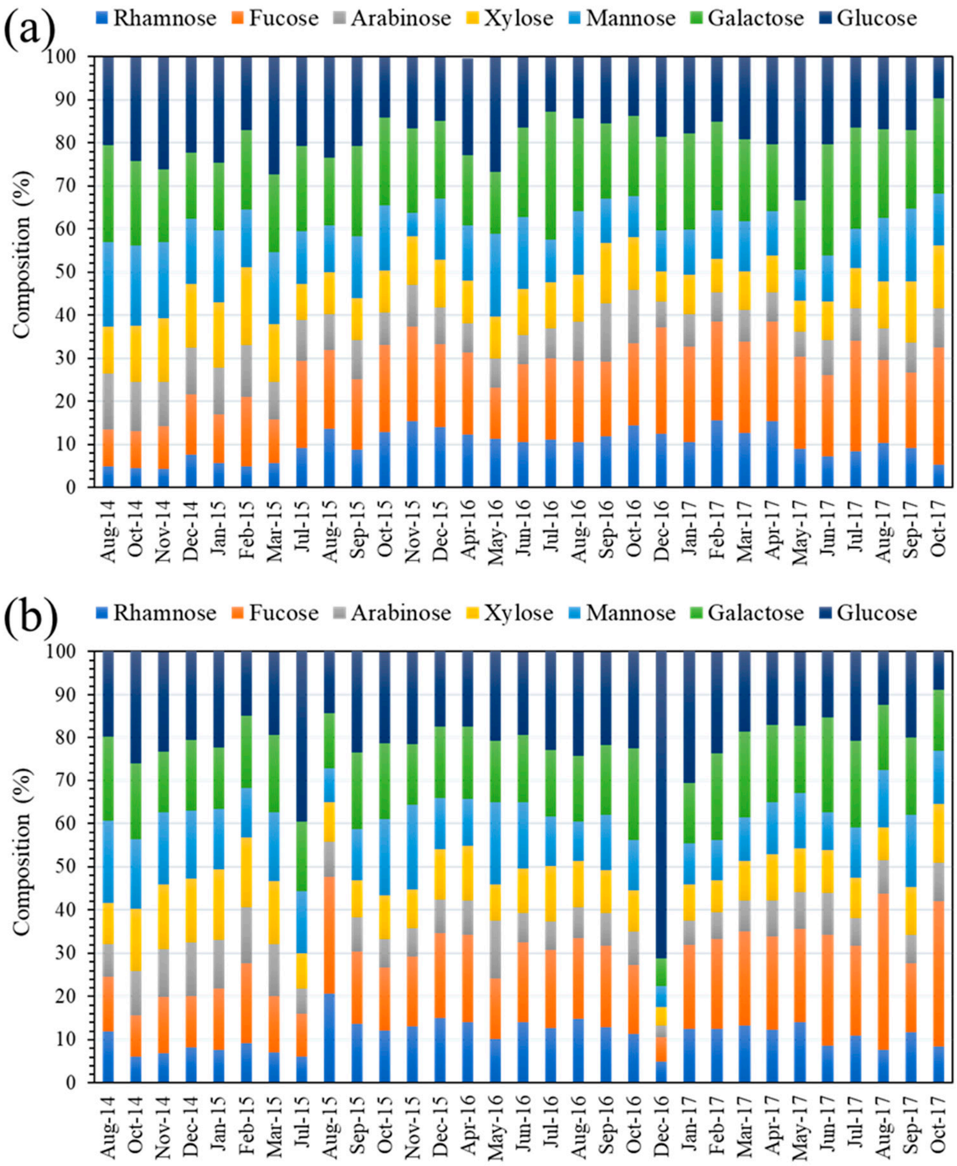

Figure S4. Neutral monosaccharides detected in the total polysaccharides collected from the lake waters at 0.5 m (a) and 90 m (b) during 2014–2017.
